# ABCB1 and ABCG2 drug transporters are differentially expressed in non-small cell lung cancers (NSCLC) and expression is modified by cisplatin treatment via altered Wnt signaling

**DOI:** 10.1186/s12931-017-0537-6

**Published:** 2017-03-24

**Authors:** M. Vesel, J. Rapp, D. Feller, E. Kiss, L. Jaromi, M. Meggyes, G. Miskei, B. Duga, G. Smuk, T. Laszlo, I. Karner, J.E. Pongracz

**Affiliations:** 1 Department of Pharmaceutical Biotechnology, School of Pharmacy, University of Pecs, Pecs, Hungary; 2 Department of Pathology, School of Medicine, University of Pecs, Pecs, Hungary; 3 Department of Microbiology and Immunology, School of Medicine, University of Pecs, Pecs, Hungary; 4 Szentagothai Research Centre, University of Pecs, Pecs, Hungary; 5 Humeltis Ltd, Pecs, Hungary; 6 Scientific Unit, Osijek University Hospital, Huttlerova 4, Osijek, HR31000 Croatia; 7 Department of Pathophysiology, Faculty of Medicine, University of Osijek, Cara Hadrijana 10, Osijek, HR31300 Croatia

**Keywords:** ABC transporters, Wnt signaling, Cisplatin, Lung cancer

## Abstract

**Background:**

Lung cancer (LC) is still the most common cause of cancer related deaths worldwide. Non-small cell lung cancer (NSCLC) accounts for 85% of all LC cases but is not a single entity. It is now accepted that, apart from the characteristic driver mutations, the unique molecular signatures of adeno- (AC) and squamous cell carcinomas (SCC), the two most common NSCLC subtypes should be taken into consideration for their management. Therapeutic interventions, however, frequently lead to chemotherapy resistance highlighting the need for in-depth analysis of regulatory mechanisms of multidrug resistance to increase therapeutic efficiency.

**Methods:**

Non-canonical Wnt5a and canonical Wnt7b and ABC transporter expressions were tested in primary human LC (*n* = 90) resections of AC and SCC. To investigate drug transporter activity, a three dimensional (3D) human lung aggregate tissue model was set up using differentiated primary human lung cell types. Following modification of the canonical, beta-catenin dependent Wnt pathway or treatment with cisplatin, drug transporter analysis was performed at mRNA, protein and functional level using qRT-PCR, immunohistochemistry, immune-fluorescent staining and transport function analysis.

**Results:**

Non-canonical Wnt5a is significantly up-regulated in SCC samples making the microenvironment different from AC, where the beta-catenin dependent Wnt7b is more prominent. In primary cancer tissues ABCB1 and ABCG2 expression levels were different in the two NSCLC subtypes. Non-canonical rhWnt5a induced down-regulation of both ABCB1 and ABCG2 transporters in the primary human lung aggregate tissue model recreating the SCC-like transporter pattern. Inhibition of the beta-catenin or canonical Wnt pathway resulted in similar down-regulation of both ABC transporter expression and function. In contrast, cisplatin, the frequently used adjuvant chemotherapeutic agent, activated beta-catenin dependent signaling that lead to up-regulation of both ABCB1 and ABCG2 transporter expression and activity.

**Conclusions:**

The difference in the Wnt microenvironment in AC and SCC leads to variations in ABC transporter expression. Cisplatin via induction of canonical Wnt signaling up-regulates ABCB1 and ABCG2 drug transporters that are not transporters for cisplatin itself but are transporters for drugs that are frequently used in combination therapy with cisplatin modulating drug response.

**Electronic supplementary material:**

The online version of this article (doi:10.1186/s12931-017-0537-6) contains supplementary material, which is available to authorized users.

## Background

Despite of the alarming statistics of lung cancer incidence and mortality rates, development of an all-around successful therapy remains elusive. Partly, as lung cancers (LC) are highly diverse and apart from small (SCLC) and non-small cell lung cancers (NSCLC) there are morphologically diverse subtypes that are frequently mixed. Additionally, their clinical characteristics and drug sensitivity also varies greatly. As there are no available targeted therapies for each variation, the treatment approaches are similar and the overall prognoses are still grim.

The 5 year survival rate of NSCLC varies from 73% in early detection (stage IA) to 3.7% at advanced metastatic disease [1, 2]. At early stages of NSCLC surgery and chemotherapy are still the choices of first line treatment [3, 4], although targeted molecular therapies are now more widely included in the treatment regimen. Targeted therapies that can extend progression free and overall survival are only available to a fraction of patients as such approaches require the presence of mutations or amplifications of specific genes most frequently KRAS, EGFR or ALK [1, 5, 6].

Unfortunately, the majority of patients are presented at advanced or even metastatic stage of the disease where surgical resection is not an option. Cisplatin based therapy can increase the survival rates in all stages but chemotherapy resistance and disease recurrence remain major issues. In metastatic disease, treatment is frequently based on the combination therapies of cisplatin or carboplatin with drugs such as paclitaxel, docetaxel, gemcitabine and vinorelbine to increase efficacy compared to single agent platinum therapy [7]. And although the use of immune modulators (e.g. Nivolumab) has become a promising route to effectively halt disease progression, their application in fast progressing tumor types require further analysis including drug interaction studies between small molecules and biological therapies [8].

Studies of influx and efflux mechanisms of drug transporters might help to evaluate effectiveness of drugs in therapy. In various studies ATP-binding cassette (ABC) transporter family members, such as ABCB1 (MDR1 or Pgp) and ABCG2 (BCRP1) are involved in drug resistance [9, 10]. While cisplatin is not an ABCB1 or ABCG2 substrate [11, 12], association studies implicated ABCG2 as a predictive factor for poor clinical outcome of advanced NSCLCs [13]. The link of ABCB1 to NSCLC chemoresistance remained inconclusive [14–16], although drugs like erlotinib, irinotecan etc. that are widely used in combination therapy of NSCLC are ABCB1 and ABCG2 substrates [17, 18].

Interestingly, it has recently been shown that TCF/LEF dependent signaling activates the ABCB1 promoter indicating that beta-catenin dependent Wnt signaling [19] is involved in ABCB1 expression [20] and therefore potentially in chemoresistance [21, 22]. The highly complex and evolutionarily conserved Wnt signaling pathway controls many developmental and tissue maintenance events via the canonical or beta-catenin dependent, and two non-canonical pathways [19]. Previous studies have shown that Wnt pathway, although rarely mutated, is differentially regulated in LC subtypes. While beta-catenin dependent signaling can be strongly down-regulated in SCLC through over-expression of inhibitory genes differential activation of the beta-catenin dependent canonical and Ca^2+^ dependent non-canonical Wnt pathways have been reported in NSCLC subtypes of AC and SCC, respectively [23, 24].

As resistance to chemotherapy is a major obstacle to successful treatment of lung cancer and resistance is strongly associated with influx and efflux drug transporters, we have theorized that the generally applied platinum-based therapy might be able to affect drug transporter expression and activity via modulation of Wnt signaling. Thus, the aim of the present study was to test the following hypotheses: (i) Expression of ABC transporters are different in AC and SCC subtypes of NSCLC patients due to the highly diverse Wnt microenvironment; and (ii) Differential expression and functionality of ABCB1 and ABCG2 are directly regulated by cisplatin modified Wnt signaling leading to altered response to second in line drugs.

## Methods

### Ethical Statement

Lung tissue samples were collected at the Department of Surgery coordinated by the Department of Pathology, University of Pecs, Hungary. The project was approved by the Ethical Committee of the University of Pecs (ETT-TUKEB 366/2015). Patients had given written consent to provide samples for research purposes. All collected samples were treated anonymously.

### Human lung samples

A total of 90 patient samples were processed, 76 adenocarcinomas and 14 squamous cell carcinomas that were collected at the Department of Surgery, assessed by a certified lung pathologist based on WHO guidelines.

### Cell culture

Primary human small airway epithelial cells (SAEC), normal human lung fibroblasts cells (NHLF) and normal human lung microvascular endothelial cells (HMVEC-L) were cultured according to the manufacturers’ recommendations (Lonza, Basel, Switzerland). Briefly, cells were thawed and cultured as monolayer in Small Airway Growth Medium (SAGM), Fibroblast Growth Medium (FGM-2) and Endothelial Growth Medium (EGM-2) respectively at 37 °C in humidified atmosphere containing 5% CO_2_. A549 lung adenocarcinoma were grown in complete DMEM containing 10% FBS and H520 lung squamous carcinoma cells (American Type Culture Collection, Rockville, MD) were cultured in complete RPMI containing 10% FBS at 37 °C in humidified atmosphere containing 5% CO_2_. Trypan blue dye exclusion test was used to assess cell viability in all culture types.

### Drugs used in the experiments

Cisplatin, Paclitaxel, Doxorubicin and Gemcitabine were all purchased from Selleck Chemicals (Selleck Chemicals, Houston, USA). Treatments were conducted at final concentrations as follows cisplatin of 29.7 μM [25], paclitaxel of 100 nM [26], doxorubicin of 100 nM [26] and gemcitabine of 100 nM [26].

### Cellular fractionation and Western-blot analysis

A549 lung adenocarcinoma cells (American Type Culture Collection, Rockville, MD) were grown in complete DMEM until reaching 70% confluence. Cells were treated for 3 h with cisplatin (Selleck Chemicals, Houston, USA) at final concentration of 29.7 μM [25]. All experiments were done in triplicates. After treatment, cells were rinsed with PBS and collected with trypsin, then proceeded with protocol for cell compartment isolation. A549 cells were used between passage number 8 to 10 and epithelial characteristics were proved by cytokeratin positivity. All the cell cultures were regularly tested for mycoplasma infection [27].

### 3D lung aggregate cultures

Aggregates containing NHLF, SAEC and HMVEC-L cells were mixed at 4:3:3 ratio, and dispensed onto poly-HEMA-coated (final concentration 20 mg/ml) 96-well plates (TPP, Sigma-Aldrich, St. Louis, USA) as described earlier [28]. The plates were centrifuged for 10 min at 600 x g at 4 °C. The final number of cells/well was 30.000. Aggregates were grown in SAGM/EGM-2/FGM-2 media at 2:2:1 ratio. After 24 h 3D aggregates were transferred on a 24 well plate (also coated with poly-HEMA) and treated with recombinant human Wnt5a at final concentration of 1 μg/ml for 72 h (Chinese Hamster Ovary Cell Line, CHO-derived Gln38-Lys380) (R&D Systems, Minneapolis, USA) [23], cisplatin (Selleck Chemicals, Houston, USA) (29.7 μM for 72 h) [25], IWR-1 (Sigma Aldrich, St. Louis, USA) (1 μM for 3 h) [23] and LiCl (Sigma Aldrich, St. Louis, USA) (10 mM for 3 h) [29]. Upon treatment they were collected in RA1 Buffer Solution for RNA isolation, into cryostat embedding medium for dissection or used for drug transporter functional test.

### RNA isolation, cDNA synthesis and qRT-PCR

3D aggregates were collected in RA1 Buffer Solution then on-column RNA isolation was performed according to the manufacturers’ protocol (NucleoSpinII RNA isolation kit, Macherey-Nagel, Düren, Germany). Total RNA from frozen human lung samples was isolated using TRI Reagent (Invitrogen, Thermo Fisher Scientific, Waltham, USA) with an additional DNase (Sigma-Aldrich, St Louis, USA) treatment. RNA concentration was measured using Nanodrop technology (Thermo Fisher Scientific, Waltham, USA).

cDNA synthesis was performed using random hexamer primers of the high capacity RNA to cDNA kit (Thermo Fisher Scientific, Waltham, USA) according to the manufacturers’ protocol.

SYBRGreen (Bioline, London, UK) real-time qRT-PCR reaction (Table 1) was set up using StepOne and StepOne Plus instruments (Thermo Fisher Scientific, Waltham, USA). For human samples, only samples with beta-actin Ct values below 22.5 were used for further evaluation. The relative quantities of different drug-transporters were calculated using the 2^-ddCt^ method. The reference gene was beta-actin for all the samples.

**Table 1:** List of gene specific primers used in qRT-PCR

Target	Accession number	Sequence
human beta-actin (ACTB)	NM_001101	for-GCGCGGCTACAGCTTCA
		rev-CTTAATGTCACGCACGATTTCC
human ABCB1	NM_000927	for-GCAGCTGGAAGACAAATACACAA
		rev-CCCAACATCGTGCACATCA
human ABCG2	NM_004827	for-AACCTGGTCTCAACGCCATC
		rev-GTCGCGGTGCTCCATTTATC
human ABCC1	NM_004996.3	for-GCTGGAGTGTGTGGGCAACT
		rev-CTGAGGCTGTGCCTGGAGAT
human ABCC2	NM_000392	for-GCAAACTGTTCTGGTGTGGA
		rev-CCAGCTCTATGGCTGCTAGA
human Wnt5a	NM_003392	for-CCTGCTCCTGACCGTCC
		rev-CAAAGCAACTCCTGGGCTTA
human Wnt7b	NM_058238	for-GTCCTGTACGTGAAGCTCGG
		rev-CGGAACTGGTACTGGCACTC

### Fluorescent staining and immunohistochemistry

Sections of 3D aggregate co-cultures were sectioned frozen and fixed with either acetone or 4% PFA (paraformaldehyde) solution (depending on the primary antibody used), and then stained by standard staining procedure. Briefly, blocking was done with 5% BSA/PBS solution; primary antibodies were diluted in the blocking solution and incubated for 1 h at room temperature. After washing the slides in PBS three times for 5 min, secondary antibody was applied for 45 min. Slides were then washed and the nuclei were stained using TO-PRO-3 (Thermo Fisher Scientific, Waltham, USA) in 1:1000 dilutions for 15 min. Slides were washed again and covered with Vectashield (Vector Laboratories, Burlingame, CA, USA) solution. Primary antibodies of mouse monoclonal anti human beta-catenin (E-5 clone, sc-7963, SantaCruz Biotechnology, Dallas, TX, USA) were diluted 1:50, while the rabbit monoclonal anti human phospho-beta-catenin (Ser675) (4176S, Cell Signaling Technology, Danvers, USA) antibody was used in 1:100 dilutions. Secondary antibodies were AlexaFluor-488 conjugated anti-rabbit and anti-mouse IgG antibodies from Thermo Fisher Scientific (Waltham, USA) diluted 1:200. The pictures were taken with Zeiss LSM 710 confocal microscope (Zeiss, Oberkochen, Germany), always first adjusting the parameters to the secondary antibody control and then inspecting the actual slides using the same settings. Images were evaluated using Fiji [30].

### Immunohystochemical staining of ABC transporters and Wnt ligands

Primary lung tissue and 3D co-cultures sections were cut using Leica CM1950 cryostat (Leica, Wetzlar, Germany) then fixed and stained with a routine IHC staining procedure using Vision Biosystems bond™ automated immunostainer (Leica, Wetzlar, Germany). Primary antibody of mouse monoclonal anti-human ABCG2 (CD338, clone 5D3, BD Biosciences, San Jose, USA) was used in 1:50 dilution and rabbit monoclonal anti-human ABCB1 (1:50, clone D3H1Q, Cell Signaling Technology, Danvers, USA). Rat monoclonal anti-human Wnt5a (clone 442625) and goat polyclonal anti-human Wnt7b antibodies were purchased from R&D Systems, (Minneapolis, USA) and used in 1:50 dilutions.

### Functionality test of drug transporters

All experiments were done using the “eFluxx-ID® Green multidrug resistance assay kit” (ENZO, Enzo Life Sciences, Farmingdale, New York, USA) according to the manufacturers’ protocol. Briefly, 3D aggregates were enzymatically digested using Accumax solution (Sigma Aldrich, St. Louis, USA) and single cell suspension were mixed with diluted drug transporter inhibitor molecules, incubated for 5 min at 37 °C, then all samples were incubated with diluted eFluxx-ID® Green dye for 30 min at 37 °C. After 30 min, propidium-iodide was added to all sample tubes and were measured immediately with FACS Canto II flow cytometer (BD, Biosciences, Immunocytometry Systems, Erembodegen, Belgium). Appropriated controls (stained and unstained) were included into procedure. The results are calculated as multidrug resistance (MDR) activity factor values (MAF).

### Cell compartment fractionation and Western-blotting

Cell compartment factions were obtained using Qproteome® Cell Compartment Kit (Qiagen, Hileden, Germany) according to the manufacturer’s recommendation. Cell compartment protein fractions were acetone precipitated. Protein content was then measured using Protein Assay Kit measured with Qubit® fluorometer (Thermo Fisher Scientific, Waltham, USA). Samples were mixed with 4× Laemmli sample buffer (BIO-RAD Laboratories, Hercules, California, USA) and heated to 90 °C for 5 min. SDS-electrophoresis was performed using Mini-Protean TGX™ Precast Gels (BIO-RAD Laboratories, Hercules, California, USA), at 125 mA current. Blotting was done for 3 h at 140 V with nitrocellulose membrane (GE Healthcare Life Sciences, Little Chalfont, UK). The membranes were blocked for 1 h using 5% milk powder in PBS solution. The primary antibody was rabbit anti-human beta-catenin (clone D10A8, Cell Signaling Technology, Danvers, USA), rabbit anti-human phospho-beta-catenin (clone D2F1, Cell Signaling Technology, Danvers, USA) in 1:1000 dilution and mouse anti-human beta-actin (clone AC-74, Sigma-Aldrich, St. Louis, USA) in 1:2000 overnight. Secondary antibody was goat anti-rabbit and anti-mouse IgG-HRP conjugate (BIO-RAD Laboratories, Hercules, California, USA) in 1:1000 dilution for 1 h. The proteins were detected using SuperSignal West Femto Maximum Sensitivity Substrate (Thermo Fisher Scientific, Waltham, USA) and documented with ImageQuant LAS4000 CCD camera system (Fujifilm, GE Healthcare Life Science, Little Chalfont, UK). Pictures were processed using Fiji [30].

### Statistical analysis

Kolmogorov-Smirnov test was used to determine the distribution of variables. Nonparametric tests were applied for the variables with no Gaussian distribution. Kruskal-Wallis test was used for effective differences among all groups. For comparing differences between groups in continuous variables with non-normal distribution Mann–Whitney-U tests were appropriate. Spearman correlation test was adequate to rank and measure the relationship between two continuous random variables. SPSS 22.0 package for Windows (SPSS Inc., Chicago, IL, USA) was applied for all statistical analyses. Differences were considered statistically significant at *P < 0.05, **P < 0.01 and ***P < 0.001.

## Results

### ABCB1 and ABCG2 transporters are differentially expressed in primary AC and SCC

**Fig. 1:**
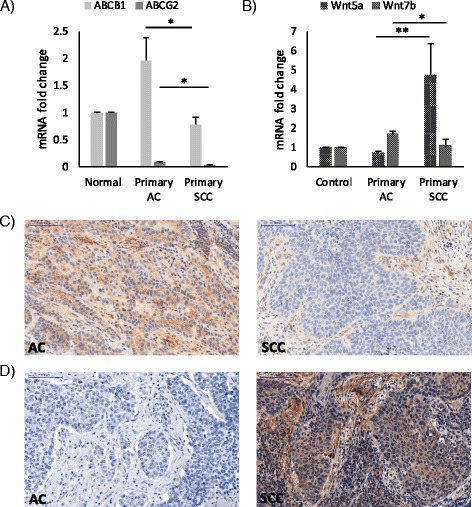
Drug transporter and Wnt microenvironment analysis of primary human AC and SCC samples. Relative mRNA expression of **a**) ABCB1 and ABCG2 drug transporters and **b**) non-canonical Wnt5a and canonical Wnt7b in primary human AC and SCC samples. mRNA expression is relative to normal, healthy lung tissue. *n* = 76 for AC, and *n* = 14 for SCC. Data are presented as mean ± SEM. Immunohistochemistry of primary AC and SCC of **c**) ABCB1 drug transporter protein staining (*n* = 5 each) and **d**) Wnt5a ligand protein staining (*n* = 5 each). Magnification is 20×, scale bar 100 μm

mRNA levels of ABCB1 and ABCG2 transporters were investigated in 90 resected, primary human lung AC and SCC tissue samples by qRT-PCR and immunohistochemistry (Fig. 1a, c). Data revealed significant differences in both ABCB1 and ABCG2 mRNA expression levels messages being higher in AC compared to SCC (Fig. 1a). Reflecting mRNA, ABCB1 protein was strongly expressed in AC but not in SCC (Fig. 1c) while ABCG2 levels were barely detectable in AC and non-detectable in SCC samples (Additional file 1: Figure S1). As the Wnt molecular background of AC and SCC differ (Fig. 1b, d), understanding any connections between the underlying molecular differences in the Wnt pathways and drug transporter induced chemoresistance can only become possible if in vitro studies are available. To model the microenvironment of the pulmonary tissue, 3D aggregate cultures were set up using primary human lung cell types HMVEC-L, SAEC and NHLF derived from commercially available healthy human donor tissues. Analysis of the aggregates revealed that ABCB1 and ABCG2 drug transporters at message levels were similar to that of primary healthy human lungs, and both transporters were expressed as active proteins (Additional file 2: Figure S2) making the model suitable for drug transporter regulation studies.

### Wnt dependent differential regulation of drug transporter expression

As differences were detected in beta-catenin dependent canonical (Wnt7b) and Ca^2+^ dependent non-canonical Wnt5a expression in AC and SCC [23] (Fig. 1b, d), respectively, the AC-like canonical Wnt pathway activation was mimicked in the lung tissue aggregate by stimulating the canonical Wnt signaling pathway using LiCl (at 10 mM final concentration) [31], the chemical activator of the beta-catenin pathway. As non-canonical pathway signals suppress the beta-catenin driven gene transcription, the SCC dependent Wnt microenvironment was simulated by the beta-catenin pathway inhibitor IWR (1 μM) [23] as well as treating the cultures with rhWnt5a (1 μg/ml final concentration).

**Fig. 2:**
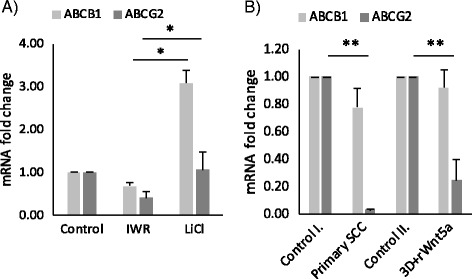
Wnt dependent differential regulation of drug transporters. Relative mRNA expression of **a**) ABCB1 and ABCG2 expression in 3D HMVEC-L-NHLF-SAEC aggregates treated with canonical Wnt pathway inhibitor, IWR and inducer, LiCl for 3 h. mRNA of treated samples is compared to untreated controls, *n* = 3 **b**) ABCB1 and ABCG2 expression in 3D HMVEC-L-NHLF-SAEC co-culture aggregates following recombinant human Wnt5a treatment shows similar pattern that SCC primary samples. mRNA expression of treated samples is compared to untreated controls, *n* = 3, mRNA expression of primary SCC samples is compared to normal lung tissue

Chemical modification of Wnt signaling revealed that inhibition of beta-catenin down-regulates both ABCB1 and ABCG2 (Fig. 2a), while activation of beta-catenin significantly up-regulates the expression of both transporters (Fig. 2a). Treatment of lung aggregate cultures with rhWnt5a reduced ABCG2 mRNA levels compared to untreated controls but had no effect on ABCB1. As ABCG2 expression in primary SCC is much lower than ABCB1 (Fig. 2b), treatment of lung aggregate cultures with rhWnt5a induced changes in drug transporter pattern in vitro that was highly similar to what was detected in primary cancer samples of squamous histology. Based on the above data we concluded that alterations in the Wnt microenvironment differentially affects both ABCB1 and ABCG2 expression.

### Cisplatin modulates the Wnt microenvironment leading to altered drug transporter activity

**Fig. 3:**
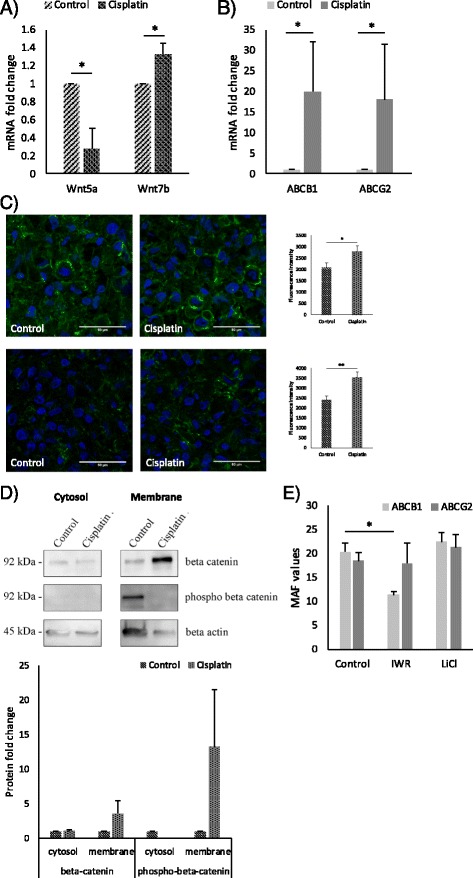
Effects of cisplatin treatment. Relative mRNA expression of **a**) Wnt5a and Wnt7b in cisplatin treated 3D HMVEC-L-NHLF-SAEC co-culture aggregates. mRNA expression of treated samples is compared to untreated controls, *n* = 3 **b**) ABCB1 and ABCG2 in cisplatin treated (29.7 μM, 3 h) 3D HMVEC-L-NHLF-SAEC co-culture aggregates. mRNA expression is normalized to beta-actin, *n* = 3 **c**) Representative images of Ser675 phosphorylated beta-catenin immune-fluorescent staining in control and cisplatin treated 3D HMVEC-L-NHLF-SAEC aggregates. Fluorescence intensity are representations of three different experiments as mean ± SEM. Scale bar 50 μm, magnification 63×. **d**) Western-blot analysis and densitometric quantification of Ser675 phosphorylated beta-catenin and beta-catenin proteins in different cell fractions of control and cisplatin treated A549 lung adenocarcinoma cell cultures, *n* = 3 **e**) functional activity of ABCB1 and ABCG2 drug transporters in 3D co-culture aggregates following canonical Wnt pathway inhibitor, IWR and inducer, LiCl treatment. Data are presented as mean ± SEM of multidrug resistance activity factor values (MAF), *n* = 3. MAF values ≤ 20 are considered as MDR negative

Cisplatin induced multidrug resistance [32] is still a major problem in cancer therapy. While cisplatin up-regulates ABCC1 and ABCC2 (Additional file 3: Figure S3) encoding multidrug resistance-associated protein 1 and 2 (MRP1 and MRP2), there is no explanation why cisplatin would affect ABCB1 or ABCG2 while not being a substrate for either transporters itself [11, 12]. To investigate, 3D lung aggregates were treated with 29.7 μM cisplatin for 72 h, then the canonical Wnt7b and the non-canonical Wnt5a message levels were measured by qRT-PCR (Fig. 3a). Cisplatin down-regulated Wnt5a (Fig. 3a), it also significantly increased the expression of the canonical Wnt7b. Simultaneously, both ABCB1 and ABCG2 mRNA levels (Fig. 3b) increased. The above data are in agreement shown in Fig. 2 where ABCB1 and ABCG2 up-regulation was detected in the presence of beta-catenin dependent Wnt pathway activation while inhibition of the same pathway resulted in reduced transporter expression (Fig. 2). To demonstrate that the beta-catenin pathway was activated by cisplatin, control and cisplatin treated 3D aggregate culture sections were stained for beta-catenin and phospho-Ser675-beta-catenin levels [33, 34]. Stabilization and therefore significant increase of beta-catenin protein as well as activated phospho-Ser675-beta-catenin was measured in samples following cisplatin treatment (Fig. 3c). Interestingly, Western blot analysis revealed a cisplatin induced cell membrane localization of active beta-catenin (Fig. 3d). To test whether modulation of beta-catenin activity correlates with transporter activity, a functional assay was performed (Fig. 3e). Although inhibition of the canonical Wnt pathway by IWR significantly suppressed ABCB1 but had no effect on ABCG2 activity, activation of the canonical pathway by 10 mM LiCl slightly but not significantly increased activity of both ABCB1 and ABCG2 transporters compared to untreated controls (Fig. 3e).

### Induction of ABC transporter mRNA levels by drugs used in combination therapy

**Fig. 4:**
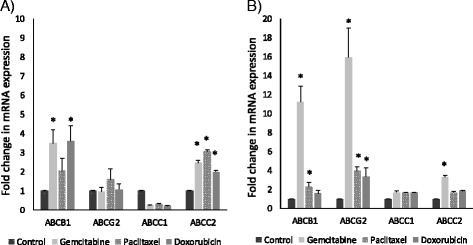
Effects of paclitaxel, doxorubicin and gemcitabine treatment. Relative mRNA expression of ABCB1, ABCG2, ABCC1 and ABCC2 were studied following treatment of **a**) 3D co-culture aggregates of adenocarcinoma cell line A549 and NHLF; and **b**) 3D co-culture aggregates of squamous cell carcinoma cell line H520 and NHLF; with 100 nM paclitaxel, 100 nM doxorubicin, 100 nM gemcitabine for 48 h. mRNA expression was normalized to beta-actin. Data are presented as mean ± SEM, *n* = 3

To test whether paclitaxel, doxorubicin and gemcitabine drugs that are frequently applied in combination therapy can affect ABC transporter transcription, 3D co-cultures were created using primary human lung fibroblasts (NHLF) and the adenocarcinoma cell line A549 or the squamous cell carcinoma cell line H520. Tissue aggregates then were treated with the above drugs at concentrations of 100 nM, respectively, for 48 h. qRT-PCR tests revealed that gemcitabine triggered a significant increase in ABCB1 and ABCC2 expression both in adeno and squamous cell carcinoma tissues (Fig. 4a, b) while paclitaxel and doxorubicin differentially initiated drug transporter transcription (Fig. 4a, b). Interestingly, while none of the above drugs increased ABCC1 transporter mRNA levels in adeno- (A549) or squamous (H520) carcinoma cell line aggregate tissues, ABCC2 were significantly up-regulated by all paclitaxel, doxorubicin and gemcitabine but only in adenocarcinoma cell line (A549) containing aggregate tissues (Fig. 4a, b).

## Discussion

Lung cancers metastasize rapidly and often become inoperable by the time of diagnosis. Cisplatin is the chemotherapeutic drug that is still used in mono- or combination therapies. Resistance to platinum agents, however, invariably develops. The developing multidrug resistance has been characterized as a multifactorial process [35] but the regulatory mechanisms leading to drug resistance are still not completely clear.

Our data revealed that ABCB1 and ABCG2 drug transporters are differentially expressed in NSCLC subtypes AC and SCC where Wnt signaling pathway activity is also different. Using a 3D lung tissue model system it was revealed that treatment of lung tissue with cisplatin up-regulates the multidrug resistance associated protein encoding ABCC1 and ABCC2 genes and via modulation of the canonical and non-canonical Wnt pathways increases ABCB1 and ABCG2 transporter expression regardless of the fact that cisplatin is not a substrate for the latter two efflux transporters [11, 12]. Inevitably, substrate specificity can also change due to mutations in drug transporter sequences [36] during disease progression. The present report provides an additional layer to the already recognized complexity of drug resistance highlighting the variable tissue microenvironment as a regulatory factor in the process. It has also become clear from the study that the characteristic and rarely mutated Wnt microenvironments in lung cancer subtypes can be mimicked and investigated in vitro. We have shown that specific drug transporter expression is characteristic to NSCLC subtypes but cisplatin can change the characteristically non-canonical (Wnt5a) microenvironment of SCC by activating the beta-catenin dependent route leading to ABCB1 and ABCG2 up-regulation. As cisplatin increased the canonical Wnt7b in the study, increase of ABCB1 and ABCG2 perhaps is not surprising as previous studies have also demonstrated beta-catenin dependent induction of ABCB1 [37, 38] in breast cancer [39, 40] and ABCG2 in colon cancer [41]. If beta-catenin was knocked down in breast cancer cells, chemosensitivity increased to doxorubicin and etoposide [39, 40]. In our study treatment with cisplatin triggered beta-catenin localization to the cellular membrane. As beta catenin is a dual function protein, regulating not just gene transcription in the canonical Wnt pathway but the coordination of cell-cell adhesion, such observation might bear additional importance. For efflux transporters to work efficiently, they need to form a functionally active complex [42–44]. If membrane structures e.g. beta-catenin-E-cadherin can stabilize lipid rafts [45] to facilitate the assembly of active drug transporter complexes, then cisplatin can increase drug efflux and lower intracellular anti-cancer drug levels in such a way that gene transcription is not even involved in the process.

Additionally, cell membrane localization of beta-catenin is normally associated with E-cadherin binding and a more differentiated epithelial phenotype. Interestingly, ABCG2 can bind to the E-box promoter region of CDH1 (E-cadherin) regulating E-cadherin expression [46]. Low levels of ABCG2 that was detected in resections of primary tumors therefore can be considered as part of the dedifferentiation process leading to low E-cadherin levels and loosened tight junction interactions facilitating cellular migration that is necessary for tumor progression.

Despite extensive studies, the precise biochemical mechanism of cisplatin induced modification of Wnt pathway activity that leads to altered ABC transporter expression and function is still not fully understood. Connection to epigenetic modification and Wnt pathway activation has been demonstrated in the literature not just for cisplatin [47–49] but for other drugs including paclitaxel, doxorubicin and gemcitabine [50, 51]. Further studies, however, needed to reveal the step-by-step biochemical mechanism and to identify molecular targets for intervention. This is particularly important as the clinical consequences are far reaching. Erlotinib and irinotecan are ABCB1 substrates while topotecan, irinotecan and tyrosine kinase inhibitors gefitinib and erlotinib are reported substrates of ABCG2 [52]. ABCG2 can also transport for example doxorubicin [53] which is used to treat brain metastasis of AC-NSCLCs [54].

## Conclusion

Drug transporters are actively involved in a complex regulatory process of drug sensitivity and resistance that is intertwined with epigenetic regulation of Wnt signaling. The present study using resected tissues of primary NSCLC subtypes and an in vitro tissue aggregate model of the lung has provided the following information: a) ABCB1 and ABCG2 drug transporters are expressed differentially in AC and SCC tissues, b) drug transporter transcription is affected differentially by cisplatin, paclitaxel, doxorubicin and gemcitabine, and c) and apart from cisplatin, none of the studied drugs increased ABCC1 message levels indicating that in vitro determination of recommended drug administration sequences can be determined to aid more favorable clinical outcome.

## Additional files

Additional file 1: Figure S1. ABCG2 and Wnt7b immunohistochemistry images of primary adeno (AC) and squamous cell carcinoma (SCC) tissues. Representative images of ABCG2 (B) and Wnt7b (B) immunochemistry of primary AC and SCC tissues (*n* = 5, each). Scale bar is 100 μm at 20× magnification and 50 μm at 40× magnification images. (DOCX 26321 kb)

Additional file 2: Figure S2. Establishing a 3D lung tissue aggregate co-culture to model drug transporter expression and activity. Relative mRNA expression of ABCB1 and ABCG2 drug transporters in A) 2D monocultures, 3D co-culture aggregates (NS: NHLF-SAEC, HNS: HMVEC-L-NHLF-SAEC), B) in 3D co-culture aggregate (HNS: HMVEC-L-NHLF-SAEC) and normal, healthy lung tissue. mRNA expression is relative to beta-actin. In 2D cultures drug transporter expressions are much lower than in the controls. These expression levels increase in 3D culture conditions and all three cell types are needed to become similar to normal lung tissue expression levels. Data are presented as mean ± SEM, *n* = 3 C) representative image of ABCG2 protein expression in 3D co-culture aggregate (HNS: HMVEC-L-NHLF-SAEC), scale bar 50 μm, magnification; D) functional activity of ABCB1 and ABCG2 drug transporters in 3D HMVEC-L-NHLF-SAEC co-culture aggregates. Data are presented as mean ± SEM of multidrug resistance activity factor values (MAF), *n* = 3. MAF values ≥ 20 are considered as active transporter function. (DOCX 655 kb)

Additional file 3: Figure S3. Effects of cisplatin in 3D lung tissue tumor cell line aggregates. Relative mRNA expression of ABCC1 and ABCC2 drug transporters of cisplatin treatment of 3D co-culture aggregates of adenocarcinoma cell line A549-NHLF (A) and (B); 3D co-culture aggregates of squamous cell line H520-NHLF (C) and (D). Data are presented as mean±SEM, *n*=3. (DOCX 55 kb)

## Abbreviations

ABC transporter: ATP Binding Cassette Family of transporters
ABCB1: ATP Binding Cassette Subfamily B (MDR/TAP) Member 1
ABCG2: ATP Binding Cassette Subfamily G (BCRP) Member 2
AC: adenocarcinoma
ALK: anaplastic lymphoma kinase
AP1: activator protein 1
ATP7A: ATPase copper transporting polypeptide alpha
ATP7B: ATPase copper transporting polypeptide beta
BSA: bovine serum albumin
CDH1: cadherin 1
CTR1 = SLC31A1: Solute Carrier Family 31 Member 1
DMEM: Dulbecco’s modified eagle medium
EGFR: epidermal growth factor receptor
EGM-2: endothelial cell growth medium
EML4: echinoderm microtubule-associated protein-like 4
FGM-2: fibroblast growth medium
GSK3β: Glycogen Synthase Kinase 3 Beta
HMVEC-L: human microvascular endothelial cells-lung
IWR: inhibitor od Wnt response
JNK = MAPK8: mitogen-activated protein kinase 8
KRAS: Kirsten Rat Sarcoma Viral Oncogene Homolog
LC: lung cancer
LEF: lymphoid enhancer binding factor
LiCl: Lithium Chloride
MAF: multidrug resistance activity factor
MDR: multidrug resistance
NHLF: normal human lung fibroblasts
NSCLC: non-small cell lung cancer
PBS: phosphate buffered saline
PCP: planar cell polarity
PFA: paraformaldehyde
PI3KCA: Phosphatidylinositol-4,5-Bisphosphate 3-Kinase, Catalytic Subunit Alpha
PKA: protein kinase A
PKC: protein kinase C
P-β-catenin: phospho beta catenin
SAEC: small airway epithelial cells
SAGM: small airway epithelial cell growth medium
SCC: squamosus cell carcinoma
SCLC: small cell lung cancer
TCF: transcription factor
WHO: World Health Organization
Wnt: Wingless-Type MMTV Integration Site Family
β-catenin: beta catenin
