# Supplementary material for: ABCB1 and ABCG2 drug transporters are differentially expressed in non-small cell lung cancers (NSCLC) and expression is modified by cisplatin treatment via altered Wnt signaling
Source: Respir Res. 2017 Mar 24;18:52. doi: 10.1186/s12931-017-0537-6 (PMC5364604; doi:10.1186/s12931-017-0537-6)
Supplement: Supplementary file 1 — ABCG2 and Wnt7b immunohistochemistry images of primary adeno (AC) and squamous cell carcinoma (SCC) tissues. Representative images of ABCG2 (B) and Wnt7b (B) immunochemistry of primary AC and SCC tissues (n = 5, each). Scale bar is 100 μm at 20× magnification and 50 μm at 40× magnification images. (DOCX 26321 kb) [file 12931_2017_537_MOESM1_ESM.docx]

**Supplementary Figure 1. ABCG2 and Wnt7b immunohistochemistry images of primary adeno (AC) and squamous cell carcinoma (SCC) tissues.** Representative images of ABCG2 (B) and Wnt7b (B) immunochemistry of primary AC and SCC tissues (n=5, each). Scale bar is 100 µm at 20x magnification and 50 µm at 40x magnification images.
