# Supplementary material for: ABCB1 and ABCG2 drug transporters are differentially expressed in non-small cell lung cancers (NSCLC) and expression is modified by cisplatin treatment via altered Wnt signaling
Source: Respir Res. 2017 Mar 24;18:52. doi: 10.1186/s12931-017-0537-6 (PMC5364604; doi:10.1186/s12931-017-0537-6)
Supplement: Supplementary file 2 — Establishing a 3D lung tissue aggregate co-culture to model drug transporter expression and activity. Relative mRNA expression of ABCB1 and ABCG2 drug transporters in A) 2D monocultures, 3D co-culture aggregates (NS: NHLF-SAEC, HNS: HMVEC-L-NHLF-SAEC), B) in 3D co-culture aggregate (HNS: HMVEC-L-NHLF-SAEC) and normal, healthy lung tissue. mRNA expression is relative to beta-actin. In 2D cultures drug transporter expressions are much lower than in the controls. These expression levels increase in 3D culture conditions and all three cell types are needed to become similar to normal lung tissue expression levels. Data are presented as mean ± SEM, n = 3 C) representative image of ABCG2 protein expression in 3D co-culture aggregate (HNS: HMVEC-L-NHLF-SAEC), scale bar 50 μm, magnification; D) functional activity of ABCB1 and ABCG2 drug transporters in 3D HMVEC-L-NHLF-SAEC co-culture aggregates. Data are presented as mean ± SEM of multidrug resistance activity factor values (MAF), n = 3. MAF values ≥ 20 are considered as active transporter function. (DOCX 655 kb) [file 12931_2017_537_MOESM2_ESM.docx]

**
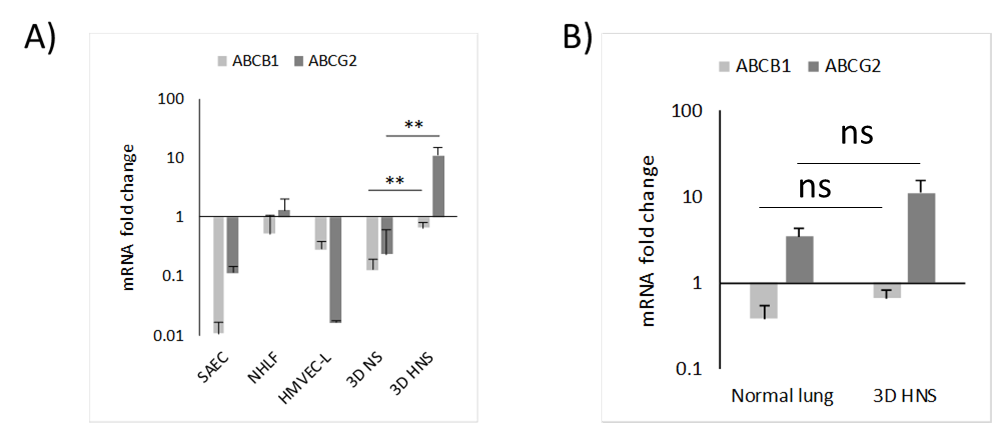
**

**
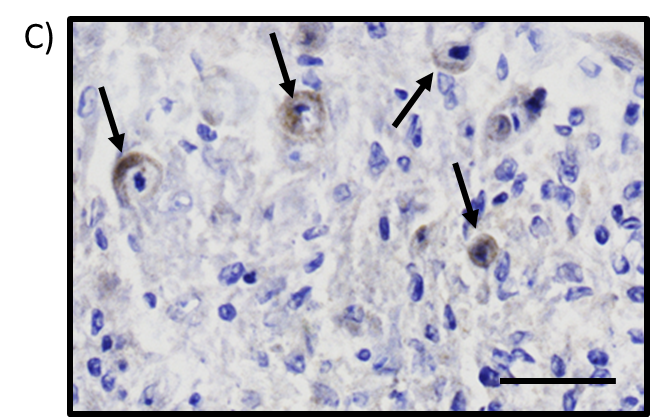
**

**
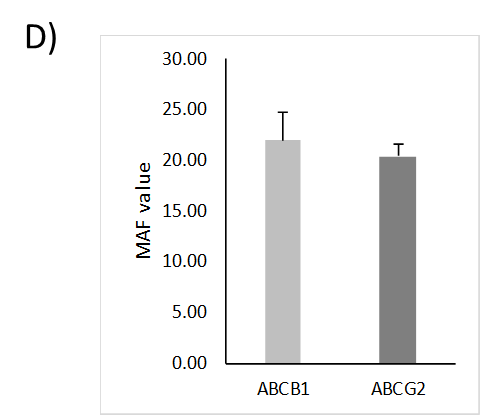
**

**Supplementary Figure 2. Establishing a 3D lung tissue aggregate co-culture to model drug transporter expression and activity.** Relative mRNA expression of ABCB1 and ABCG2 drug transporters in A) 2D monocultures, 3D co-culture aggregates (NS: NHLF-SAEC, HNS: HMVEC-L-NHLF-SAEC), B) in 3D co-culture aggregate (HNS: HMVEC-L-NHLF-SAEC) and normal, healthy lung tissue. mRNA expression is relative to beta-actin. In 2D cultures drug transporter expressions are much lower than in the controls. These expression levels increase in 3D culture conditions and all three cell types are needed to become similar to normal lung tissue expression levels. Data are presented as mean±SEM, n=3 C) representative image of ABCG2 protein expression in 3D co-culture aggregate (HNS: HMVEC-L-NHLF-SAEC), scale bar 50 µm, magnification; D) functional activity of ABCB1 and ABCG2 drug transporters in 3D HMVEC-L-NHLF-SAEC co-culture aggregates. Data are presented as mean±SEM of multidrug resistance activity factor values (MAF), n=3. MAF values ≥ 20 are considered as active transporter function.
