# Supplementary material for: ABCB1 and ABCG2 drug transporters are differentially expressed in non-small cell lung cancers (NSCLC) and expression is modified by cisplatin treatment via altered Wnt signaling
Source: Respir Res. 2017 Mar 24;18:52. doi: 10.1186/s12931-017-0537-6 (PMC5364604; doi:10.1186/s12931-017-0537-6)
Supplement: Supplementary file 3 — Effects of cisplatin in 3D lung tissue tumor cell line aggregates. Relative mRNA expression of ABCC1 and ABCC2 drug transporters of cisplatin treatment of 3D co-culture aggregates of adenocarcinoma cell line A549-NHLF (A) and (B); 3D co-culture aggregates of squamous cell line H520-NHLF (C) and (D). Data are presented as mean±SEM, n=3. (DOCX 55 kb) [file 12931_2017_537_MOESM3_ESM.docx]

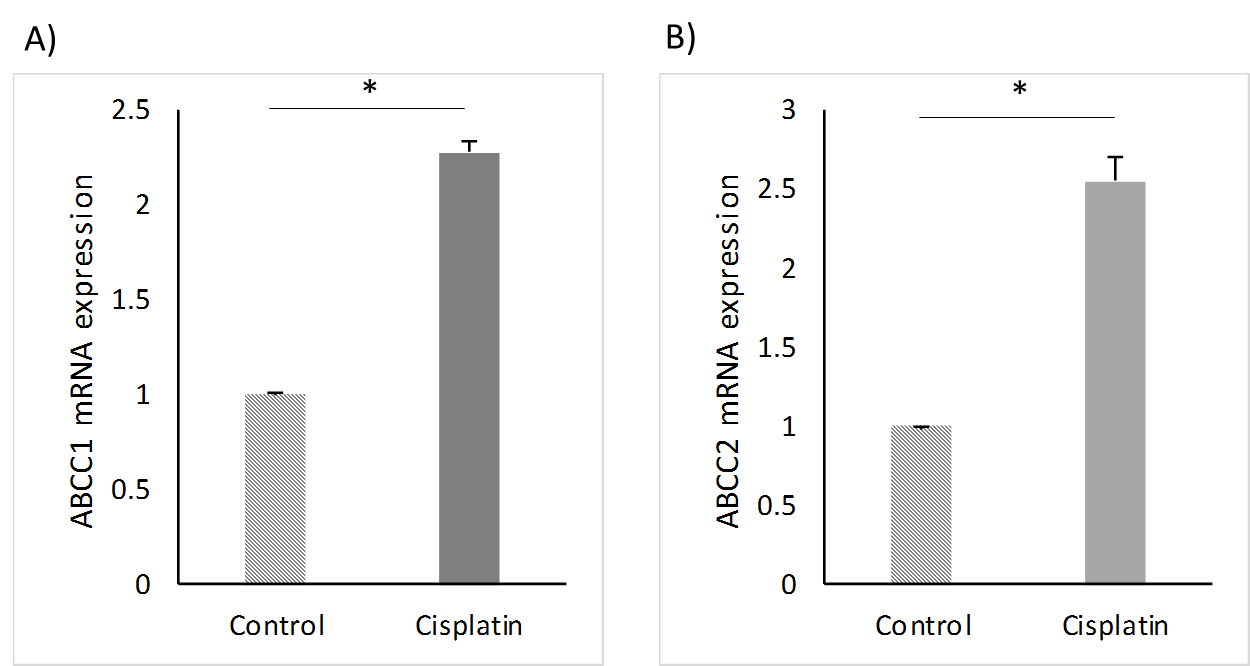


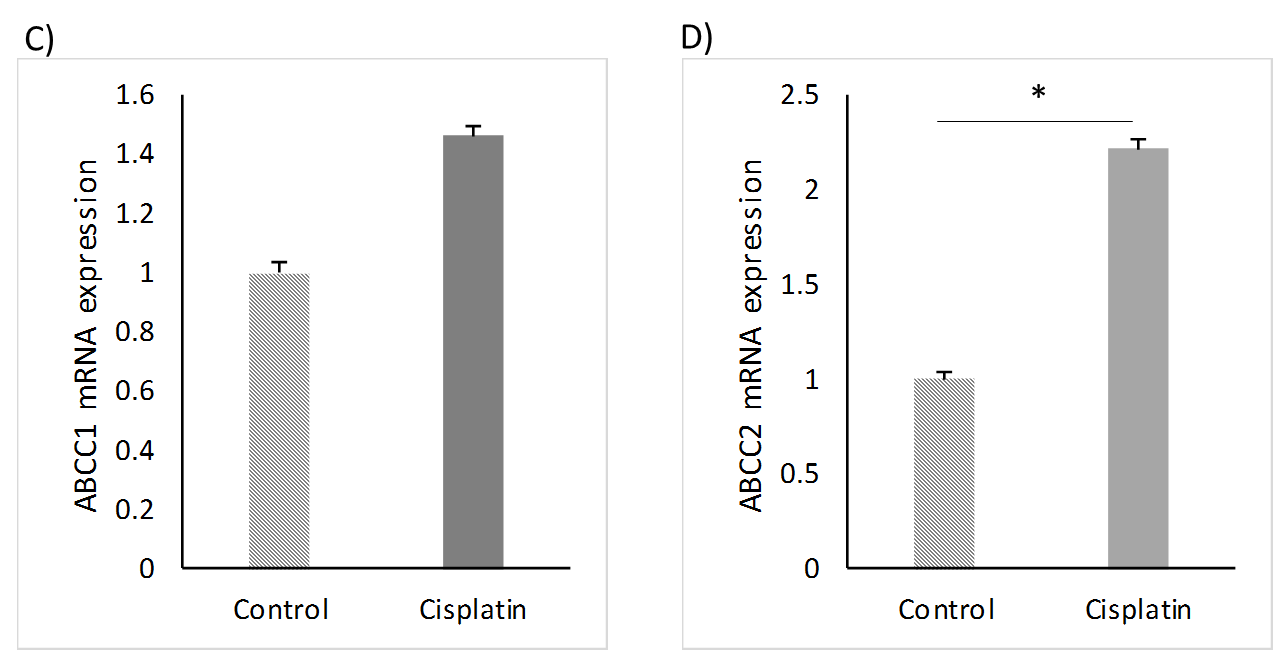


**Supplementary Figure 3. Effects of cisplatin in 3D lung tissue tumor cell line aggregates.** Relative mRNA expression of ABCC1 and ABCC2 drug transporters of cisplatin treatment of 3D co-culture aggregates of adenocarcinoma cell line A549-NHLF (A) and B)); 3D co-culture aggregates of squamous cell line H520-NHLF (C) and D)). Data are presented as mean±SEM, n=3.
